# A Rare Cause of Colonic Obstruction “Colonic Intussusception”: Report of Two Cases

**DOI:** 10.1155/2015/465374

**Published:** 2015-03-10

**Authors:** Tayfun Yoldas, Avni Can Karaca, Safak Ozturk, Mutlu Unver, Cemil Calıskan, Mustafa Ali Korkut

**Affiliations:** ^1^Department of General Surgery, Ege University Faculty of Medicine, Izmir, Turkey; ^2^Department of General Surgery, Izmir University, Medical Park Izmir Hospital, Izmir, Turkey

## Abstract

Colocolic intussusceptions are rare clinical entities in adults and almost always caused by a leading lesion which often warrants resection. Mostly being malignant, the leading lesions are rarely benign lesions where intraluminal lipomas are the most frequent among them. Most adult intussusceptions require surgical resection owing to two major reasons: common presence of a leading lesion and significantly high risk of malignancy—reaching as high as 65% regardless of the anatomic site—of the leading lesion. Resection of the affected segment is usually the treatment of choice, since preoperative diagnosis of the lesion is usually ineffective and most leading lesions are malignant. This paper represents two cases of adult colocolic intussusception caused by intraluminal lipomas with a brief review of the literature.

## 1. Introduction

An intussusception is by definition the insertion of a proximal segment of gastrointestinal (GI) tract into the lumen of subsequent segment in a telescopic fashion [1]. Although common in pediatric population, intussusception in adults is rare, causing only 1–5% of all bowel obstructions and 5% of all intussusceptions [1]. Usually a lead point is present in 70–90% of adult intussusceptions in contrast to 90% of idiopathic cases in pediatric population [2, 3]. There are also some major contrasts between the symptoms of pediatric and adult intussusceptions. Intussusception most frequently presents with intermittent colicky pain, abdominal mass, and passage of dark clots mixed with mucus in children; however it presents with nonspecific symptoms in acute, subacute, and chronic fashion in adults [4, 5]. This often leads to delays in diagnosis in adults and definitive diagnosis is commonly achieved in the operating room.

Most adult intussusceptions require surgical resection owing to two major reasons: common presence of a leading lesion and significantly high risk of malignancy—reaching as high as 65% regardless of the anatomic site—of the leading lesion [3, 4, 6]. Among these leading lesions lipomas of the colon emerge as a rare clinical entity with a reported incidence between 0.2% and 4.4% [7]. Though being rare, colonic lipomas are the most common benign nonepithelial tumors found in the gastrointestinal tract and are the third commonest tumors after hyperplastic and adenomatous polyps [8].

Hereby, we present two cases of adult colocolic intussusceptions owing to intraluminal lipomas of the colon of different anatomic sites with a brief review of the literature.

## 2. Case Presentation

### 2.1. Case 1

A 53-year-old male patient without any known history of chronic comorbidities or surgery presented with one-month history of upper abdominal discomfort and meteorism accompanied by seldom vomiting. The complaints were reported to appear roughly two hours after meals and lasted for hours. With an initial diagnosis of biliary colic and dyspepsia he was examined with abdominal ultrasonography, which revealed a pseudo kidney image in the transverse colon and showed no other abnormalities.

**Figure 1:**
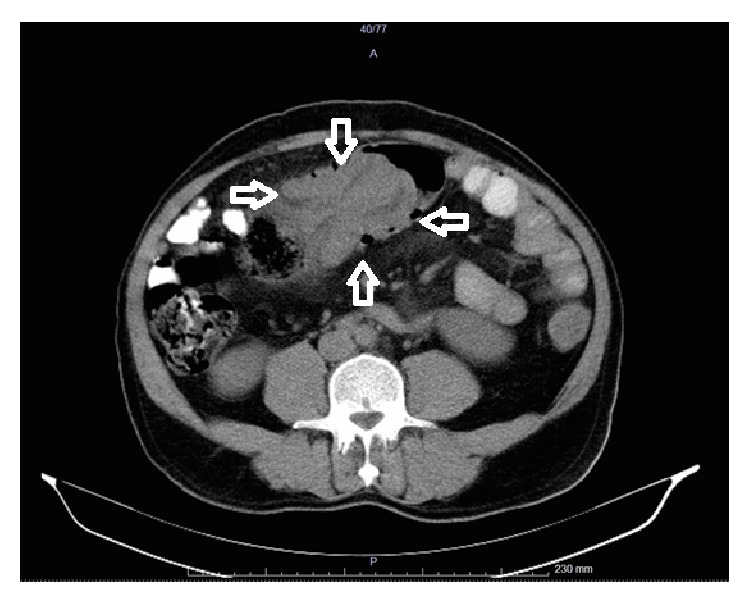
Tomographic view of the intussuscepted transverse colon.

Further examination with abdominal tomography revealed diffuse thickening of the transverse colon wall along a 9 cm segment and colocolic intussusception (Figure 1). Endoscopic examination clearly visualized intraluminal fatty lesion leading to intussusception in the distal segments of the transverse colon accompanied with nonnecrotizing intussusception.

Under these findings the patient underwent left hemicolectomy with end-to-end anastomosis. The postoperative course was uneventful and the patient was discharged on the 6th postoperative day.

Histopathologic examination of the specimen revealed a lipomatous lesion with a diameter of 5 cm of pure benign nature and 11 lymph nodes without any signs of malignancy.

### 2.2. Case 2

**Figure 2:**
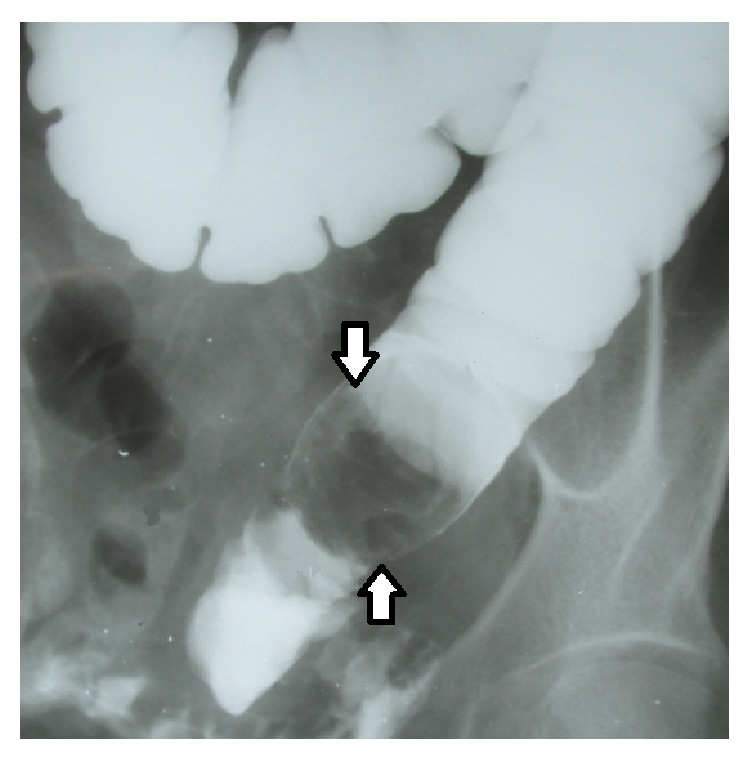
Endoluminal filling defect in barium enema study.

A 51-year-old female patient without any known history of chronic comorbidities or surgery presented with progressive distention for over 20 days accompanied by vomiting and vague pain in the left lower quadrant of the abdomen. She was examined with a barium enema study revealing an intraluminal mass in the sigmoid colon, before admission (Figure 2).

She was further examined with colonoscopy again revealing an intraluminal mass accompanied by signs of intussusception.

She underwent sigmoid resection with an end-to-end colorectal anastomosis and did well on the postoperative course. She was discharged on the 4th postoperative day.

Histopathologic examination of the specimen revealed a polipoid lesion of 4 cm in diameter with slight ulceration consisting of pure fatty cells without any signs of malignancy. Examination of 9 lymph nodes again showed no signs of malignancy.

## 3. Discussion

Intussusception is a very rare cause of intestinal obstruction in adults constituting only 5% of all bowel obstructions [1]. Almost always owing to a leading lesion in adults, intussusceptions of small bowel tend to have benign leading points when compared to those in the colon [1, 3, 4, 6]. However, being rare (35%), there are also benign lesions of the colon that can lead to intussusceptions such as endoluminal lipomas [3, 4, 6, 9]. Our cases were of that rare group with nonspecific abdominal symptoms.

Since adult intussusceptions are rare clinical entities, a high index of clinical suspicion is essential in diagnosis. Contrary to pediatric intussusceptions, ultrasound imaging is rarely useful in adult population. With colonoscopy still being the golden standard of diagnosis, computerized tomography proved to be the most useful diagnostic method in adult intussusceptions, usually revealing a smooth, well demarcated sausage shaped mass [10].

Lipomas of the colon are rare tumors of mesenchymal origin, composed of well-differentiated adipose tissue, that rarely become symptomatic and are usually detected incidentally [7, 9]. They usually arise from the mesenchyme rich submucosa, occasionally extending into the muscularis propria. Up to 10% of them are reported to be subserosal but transmural lodging is extremely rare [11]. Most common location for colonic lipomas is ascending colon and cecum [12]. However, our patients varied from the data from the literature by means of localization. Considering the high incidence of malignancy regardless of anatomic site (65%) [3, 4, 6] for the leading points of intussusception in the colon, we believe the wisest option is a proper colon resection for these kinds of patients. Endoscopic resection options are also suggested in the literature but indications are limited to small (less than 5 cm in diameter) and asymptomatic lipomas, keeping the risk of bleeding and perforation in mind [13, 14]. Colotomy and limited resections are other less favorable options offered in the literature since accurate perioperative diagnosis is difficult especially when the lesion is large in size and with ulceration, thus surgical colon resection is suggested as the treatment of choice for large, symptomatic lesions where malignancy cannot be completely excluded [15].

In conclusion, colocolic intussusceptions in adults are rare clinical entities with vague and nonspecific symptoms. Almost always they are caused by a leading lesion, which is commonly malignant. Lipomas of the colon are rarely encountered as the leading point but they should be resected nonetheless since malignancy cannot be totally excluded in large lesions.
